# Attention-Aware Graph Neural Network Modeling for AIS Reception Area Prediction

**DOI:** 10.3390/s25196259

**Published:** 2025-10-09

**Authors:** Ambroise Renaud, Clément Iphar, Aldo Napoli

**Affiliations:** 1 Centre for Research on Risks and Crises, Mines Paris-PSL, F-06904 Sophia Antipolis, France; ambroise.renaud@minesparis.psl.eu; 2 UMR 6554 LETG, University of Western Brittany (UBO), F-29200 Brest, France; clement.iphar@univ-brest.fr

**Keywords:** ship tracking, graph neural networks, AIS reception prediction, radio wave propagation modeling, data-driven approach

## Abstract

Accurately predicting the reception area of the Automatic Identification System (AIS) is critical for ship tracking and anomaly detection, as errors in signal interpretation may lead to incorrect vessel localization and behavior analysis. However, traditional propagation models, whether they are deterministic, empirical, or semi-empirical, face limitations when applied to dynamic environments due to their reliance on detailed atmospheric and terrain inputs. Therefore, to address these challenges, we propose a data-driven approach based on graph neural networks (GNNs) to model AIS reception as a function of environmental and geographic variables. Specifically, inspired by attention mechanisms that power transformers in large language models, our framework employs the SAmple and aggreGatE (GraphSAGE) framework convolutions to aggregate neighborhood features, then combines layer outputs through Jumping Knowledge (JK) with Bidirectional Long Short-Term Memory (BiLSTM)-derived attention coefficients and integrates an attentional pooling module at the graph-level readout. Moreover, trained on real-world AIS data enriched with terrain and meteorological features, the model captures both local and long-range reception patterns. As a result, it outperforms classical baselines—including ITU-R P.2001 and XGBoost in F1-score and accuracy. Ultimately, this work illustrates the value of deep learning and AIS sensor networks for the detection of positioning anomalies in ship tracking and highlights the potential of data-driven approaches in modeling sensor reception.

## 1. Introduction

Ship tracking is a key component of naval operations, encompassing the ability to effectively monitor and understand maritime activities that may affect safety, security, economic interests, or the environment. One of the most widespread technologies supporting this capability is the Automatic Identification System (AIS). The International Maritime Organization (IMO) implemented the AIS in the early 2000s. The AIS is a transponder-based communication system enabling vessels to broadcast key navigational data—such as position, speed and identity—to nearby ships and coastal monitoring stations. Introduced in the 1990s, its primary objective was to facilitate automatic information exchange to enhance maritime safety and improve traffic management efficiency [1].

The AIS operates over two Very High Frequency (VHF) bands centered around 162 MHz and uses a time-slotted protocol to manage transmissions. It combines a VHF transceiver with a Global Navigation Satellite System (GNSS) module to determine and broadcast vessel positions. Two classes of AIS exist: Class A, which is mandatory, required for large commercial and passenger vessels, and Class B, which is non-mandatory, a lower-cost version for smaller vessels.

Although the AIS is widely adopted and generally reliable, it remains vulnerable to errors, falsification, and malicious activities, such as spoofing or jamming [2]. The quality of AIS transmissions depends not only on device characteristics and the system’s power level—typically for Class A devices, 1 W in reduced mode when the vessel is docked and 12.5 W in normal mode when the vessel is at sea—but also on environmental and atmospheric conditions [3]. Interference due to congestion [4], jamming and falsification [2], or abnormal atmospheric conditions may affect signal quality, reducing the receiver’s ability to correctly interpret transmissions and potentially leading to security incidents, collisions, or undetected illegal activity [5]. Other disruptions may also arise from technical issues such as hardware malfunctions (e.g., faulty cables, misaligned antennas) [6], or operational failures like outdated AIS updates, improper system initialization and the misuse of features like silent mode, which limit the transmission range [7,8]. Consequently, many authors emphasize the importance of received signal strength studies for enhancing ship tracking [9,10,11].

While most transmissions occur in the line-of-sight (LoS) to coastal AIS stations, certain cases show reception beyond the LoS. Reception areas are defined as zones where coastal stations and ships can always receive transmitted messages [12,13]. At 162 MHz, several propagation phenomena influence reception areas. Terrain-induced effects such as diffraction are significant [14,15], along with tropospheric scattering, atmospheric refraction and ducting, which are driven by variations in atmospheric refractivity [16,17,18,19,20]. Multipath propagation due to reflection and scattering, especially diffuse reflection, also affects reception [21,22,23]. These phenomena fall under the category of anomalous propagations [24]. It is important to distinguish between cases of genuinely anomalous propagations and the potential falsification of AIS messages that can affect ship tracking and behavior analysis. Incorrect localization of a vessel in the ship tracking process will lead to misestimated trajectories and misinterpreted vessel behavior. It is therefore essential to incorporate advanced analysis of anomalies in the data analyzed and in the detection of associated behaviors [25,26,27]. For this purpose, we propose to predict the reception area of an AIS sensor in order to verify and validate the possible geographical origin of an AIS message and thus the location of the emitting ship.

Radio wave propagation modeling has an extensive history, with foundational contributions that span numerous domains such as telecommunications, radar and broadcasting. Over time, three principal categories of models have emerged, each offering distinct perspectives and trade-offs:

- Deterministic models: Grounded in electromagnetic theory, these aim to provide accurate predictions by solving wave equations, such as the Parabolic Equation (PE) method [28,29,30,31], FSPL and LoS-based models.
- Empirical models: These use statistical fits from measurement campaigns, like the ITU-R P.1546 [32], Okumura [33,34] and Egli [35] models.
- Semi-empirical models: These combine theoretical insight with empirical adjustments for practical use, such as ITU-R P.452 [36], P.1812 [37], P.2001 [38], COST 231-Hata [39] and the Longley–Rice (ITM) model [40].

While physically grounded and interpretable, these models often face challenges in generalizing across highly dynamic or heterogeneous environments, particularly when conditions deviate from their underlying assumptions. Moreover, they require extensive calibration and access to fine-grained environmental inputs such as atmospheric profiles or terrain descriptors, which are not always available or reliable in operational contexts. These limitations motivate the exploration of alternative approaches that can adapt more flexibly to observational data and operate under less constrained input conditions.

Recent developments in environmental data availability and machine learning have enabled new, data-driven alternatives [41,42]. Classical machine learning models—such as decision trees, support vector machines, random forests and gradient boosting—have demonstrated the ability to learn predictive functions directly from empirical observations without the need to explicitly encode the underlying physical propagation mechanisms [43,44,45,46]. These models are generally faster to train, require less domain-specific tuning and are easier to interpret than deep learning (DL) approaches. However, their performance may degrade in complex settings involving high-dimensional or structured inputs. This has led to increasing interest in deep learning techniques that are capable of learning hierarchical and spatially-aware representations directly from raw or minimally processed data. More recently, deep learning methods, including Multi-Layer Perceptrons (MLPs), Convolutional Neural Networks (CNNs) and graph neural networks (GNNs), have shown promise in modeling complex propagation environments [47,48,49,50].

In the context of AIS propagation, machine learning (ML)-based approaches have begun to emerge, leveraging environmental and observational data to predict reception quality and signal range [51,52,53]. These models offer a flexible complement to traditional methods and may be particularly valuable in scenarios involving interference, complex terrain, or anomalous conditions.

Among deep learning approaches, GNNs offer a particularly compelling framework for modeling spatial propagation phenomena such as AIS propagation. Their capacity to perform message passing across nodes allows them to capture both local interactions—such as terrain effects—and longer-range dependencies influenced by atmospheric conditions. Moreover, GNNs support inductive generalization, enabling the model to predict on previously unseen graph topologies, which is essential in dynamically evolving or geographically diverse maritime contexts. These properties make GNNs a natural fit for AIS signal modeling, where spatial context, topology and environmental structure are key predictive factors.

In this paper, we propose a GNN-based modeling approach to predict AIS reception areas as a function of environmental variables. This method aims to contribute to the broader goal of enhancing ship tracking.

The remainder of the paper is structured as follows: Section 2 presents deep learning techniques for radio wave propagation modeling. Section 3 describes the dataset, preprocessing steps and modeling approach. Section 4 reports on model training, evaluation and comparison with traditional models. Section 5 discusses the limitations and suggests future research directions.

## 2. Related Works

Machine learning encompasses a variety of algorithmic paradigms aimed at learning patterns from data. It is commonly divided into categories such as supervised learning, unsupervised learning and reinforcement learning, each corresponding to different types of tasks and data availability. In particular, supervised learning applies when labeled data are available and the objective is to learn a mapping from inputs to known outputs. Given a set of input features and corresponding labels, the model learns to approximate a function that maps inputs to outputs. In the context of radio wave propagation, supervised learning can be applied to predict signal strength, reception probability, or coverage classification, using historical signal measurements and environmental variables as input. The success of supervised learning methods depends on the quality of the training data and the representational capacity of the model [54].

Deep learning refers to a class of machine learning methods based on deep artificial neural networks, which are composed of multiple layers that successively transform input data into more abstract representations [55]. These models are capable of automatically learning complex, nonlinear relationships without the need for handcrafted features. Common architectures include MLPs, CNNs and Recurrent Neural Networks (RNNs), each tailored for specific data structures such as tabular, spatial, or temporal inputs [41]. Deep learning models have demonstrated strong performance in modeling radio propagation, especially in complex environments where traditional or regression-based models are limited. These models can automatically extract relevant features from high-dimensional input data, such as environmental characteristics, terrain data and spatial maps, without the need for predefined functional forms [47]. Deep learning provides a robust and flexible framework for propagation modeling in real-world conditions. This is also true for AIS propagation modeling, where deep learning methods have already shown promising results. In a recent study, ref. [53] proposed a deep learning approach that leverages both meteorological data and historical AIS detection ranges to forecast AIS sensor performance. Using MLP and Long Short-Term Memory (LSTM) models, their system predicts the spatial extent of AIS reception 24 h in advance, based on 72 h of past data. Those results demonstrate high accuracy, highlighting the relationship between environmental data and received signal strength.

These models typically rely on regular grid structures or sequential inputs. However, many physical systems—including AIS propagation—naturally exhibit relational structures that can be represented as graphs. Graphs are mathematical structures used to model relationships between entities. A graph is composed of nodes and edges that connect pairs of nodes. In the context of geospatial or physical systems, nodes can represent emitters, receptors or scatters and edges can encode relationships such as functional dependencies or interactions [56]. An emerging architecture that leverages graph representations is the GNN, which extends deep learning by operating directly on graph structures [57]. Through message-passing mechanisms, each node updates its representation by aggregating information from its neighbors across the graph. This makes GNNs particularly suitable for wireless network applications, where spatial dependencies and dynamic topologies naturally lend themselves to graph-based representations [58,59]. Ref. [59] offers a broad overview of GNN applications in wireless networks. The authors highlight how various graph construction strategies can be tailored to encode physical constraints and relational priors. Their findings further support the idea that GNNs provide a versatile and scalable solution for modeling signal behavior across diverse propagation environments. Recently, ref. [49] demonstrated the effectiveness of GNNs for predicting radio coverage maps using real-world 4G measurements. Their model represents spatial cells as nodes in a graph, with edges encoding both proximity and ray-tracing-inspired directional links to model realistic signal propagation.

Together, these studies reinforce the potential of GNN-based architectures for applications such as AIS reception prediction, where spatial, environmental and directional factors must be jointly considered within a structured, relational framework. Despite the increasing use of GNNs in wireless coverage prediction and general signal modeling, to the best of our knowledge, no prior work has specifically explored their application to AIS propagation modeling.

This last remark highlights a gap in the current literature and motivates our proposed approach. Thus, the main objective of this research is to develop and evaluate a graph neural network-based framework for predicting AIS reception areas as a function of environmental and geographic variables. By addressing the limitations of classical physics-based and machine learning models, our goal is to provide a scalable and data-driven methodology that enhances ship tracking reliability and supports the detection of positioning anomalies in maritime monitoring. Section 3 details the dataset construction, graph formulation and GNN architecture developed to address this challenge.

## 3. Materials and Methods

This section presents the overall methodology developed for AIS reception prediction using graph neural networks. Transformers power modern large language models (LLMs) by using attention to select and integrate relevant context across long sequences [60,61]. Similar principles have informed graph learning. The proposed architecture combines SAmple and aggreGatE (GraphSAGE) convolutions with an LSTM-based Jumping Knowledge (JK) mechanism, where layer contributions are adaptively controlled through attention coefficients computed by Bidirectional Long Short-Term Memory (Bi-LSTM) [62]. Attention also enters at the graph-level readout, where an attentional aggregation module provides data-driven pooling weights [63]. For comparison, we further evaluate a Graph Attention Network (GAT) [64], highlighting how attention may operate at different levels: across depths (JK), at readout (pooling), and across neighborhoods (GAT). While dedicated graph-transformer architectures now integrate attention globally across all nodes and layers [65], our design shows that combining simpler attention mechanisms already yields a transformer-aligned pathway for expressive and efficient graph representations.

This design captures both local and global spatial dependencies, supports inductive learning, and is well-suited to dynamic maritime environments. We then introduce the data sources used to model signal reception, including AIS reception measurements and contextual variables. These datasets are preprocessed and integrated to build a comprehensive input space for graph-based learning. Finally, we detail the graph construction pipeline and feature engineering process, which translates the gridded data into a structured representation that is suitable for GNNs. Each graph is generated from a transmitter-centric cone and encodes the spatial relationships necessary for classification.

### 3.1. Proposed Neural Network Architecture

**Figure 1:**
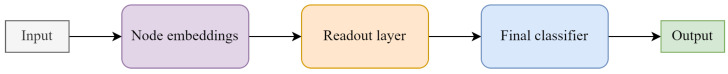
General framework for graph classification.

We adopt a graph classification framework, where each input instance is a directed graph representing a localized AIS reception scenario. The task is to predict a binary label indicating whether a given subgraph corresponds to a region of positive AIS signal reception. The general framework adopted for our graph classification problem is presented in Figure 1 [66].

**Figure 2:**
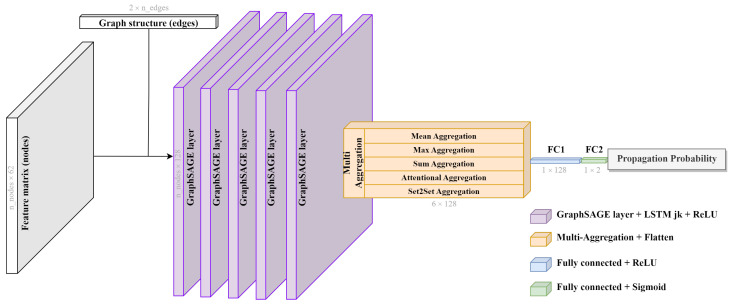
Detailed architecture of the proposed GNN for AIS reception prediction.

Our architecture—detailed in Figure 2—is built upon GraphSAGE, a neighborhood aggregation method that is well-suited for inductive learning tasks [58,67]. GraphSAGE allows the model to generalize to unseen graph topologies by learning functions that aggregate and transform information from node neighborhoods. Unlike transductive methods, GraphSAGE does not require the full graph structure at training time, which aligns well with our setting where graphs are generated dynamically for different spatiotemporal instances.

To enhance expressivity, we integrate a Jumping Knowledge mechanism based on a weighted summation scheme [62]. Instead of relying solely on the last GNN layer, this approach aggregates node representations from all intermediate layers through attention weights obtained from a bi-directional LSTM [68], as shown in Equation (1).

(1) $$h_{v}^{\mathrm{JK}}=\sum_{t=1}^{L}\alpha_{v}^{\left(t\right)}h_{v}^{\left(t\right)},$$

where $h_{v}^{\left(t\right)}$ is the embedding of node *v* at layer *t*, *L* is the total number of layers, and the attention scores $\alpha_{v}^{\left(t\right)}$ are learned in a node-adaptive way from the Bi-LSTM.

This design allows the model to dynamically adjust its receptive field and integrate multi-scale spatial patterns, which is important given the variability in graph size and topology across instances.

After obtaining the final node embeddings, a graph-level representation is computed using a multi-aggregation readout layer. Instead of relying on a single pooling strategy, we concatenate the outputs of five distinct aggregation functions:

- Mean aggregation, used to capture the distribution [69];
- Max aggregation, used to identify representative elements [69];
- Sum aggregation, used to learn structural graph properties [69];
- Attentional aggregation weights node contributions via a trainable gate network [63];
- Set2Set aggregation models higher-order dependencies using a recurrent global attention mechanism [70].

This combination allows the model to preserve both local and global characteristics of the graph structure, increasing the robustness and informativeness of the representation [71,72,73].

The resulting pooled feature vector is fed into a two-layer Multi-Layer Perceptron, which serves as the final classifier. The MLP applies a non-linear transformation followed by a sigmoid activation function to output a probability $\hat{y}\in \left[0,1\right]$, representing the predicted likelihood of AIS signal reception for the input subgraph.

The design of our GNN architecture is not only data-driven but also inspired by the physical nature of radiowave propagation.

The graph structure itself models the spatial domain through which AIS signals propagate. Nodes correspond to discretized geographical units and directed edges represent potential propagation paths. The edge directionality (transmitter (Tx) → receiver (Rx)) mimics the physical direction of signal emission and accounts for geometric attenuation with distance and orientation.

Neighborhood aggregation in GraphSAGE approximates local wavefront diffusion. By averaging or summarizing node features from immediate neighbors, the GNN mimics how environmental factors at adjacent regions influence the strength of the signal at a given point [49].

The cone-based subgraph extraction aligns with the concept of radiation patterns and ray cones in radio physics [74], limiting the spatial domain to areas that are most likely to affect or be affected by transmission. This geometric prior enhances the relevance of selected nodes and reduces noise.

Further, the attention-based aggregation allows the model to weigh spatial zones with greater influence [64]—analogous to focusing on areas with less environmental loss or more favorable meteorological conditions. Similarly, the Set2Set module allows the global context to be incorporated, similar to long-range propagation influenced by atmospheric ducting or anomalous refraction layers.

Together, these components form a neural architecture that not only learns from data but also reflects physical assumptions, improving alignment with domain knowledge.

#### Model Variants and Alternatives

Several architectural alternatives were considered but not retained in the final model, based on theoretical limitations and preliminary experimentation.

We choose GraphSAGE over attention-based GNNs such as GAT due to its scalability and robustness [75].

In the context of Jumping Knowledge, we test mean aggregation, concatenation and LSTM strategies. JK-Mean computes a uniform average of layer-wise node representations, as defined in Equation (2):

(2) $$h_{v}^{\mathrm{JK}-\mathrm{Mean}}=\frac{1}{L}\sum_{\ell =1}^{L}h_{v}^{\left(\ell \right)}$$

while simple and efficient, this averaging tends to dilute signals from deeper layers.

JK-Concat, on the other hand, stacks the embeddings from all layers, as expressed in Equation (3):

(3) $$h_{v}^{\mathrm{JK}-\mathrm{Concat}}=h_{v}^{\left(1\right)}\;∥\;\ldots \;∥\;h_{v}^{\left(L\right)}$$

this preserves the richness of multi-scale representations but at the cost of increased model complexity.

Moreover, JK-LSTM dynamically adjusts the combination of embeddings based on the structural context, allowing it to capture spatial hierarchies without excessive oversmoothing [62]. Empirically, JK-Mean produces unstable validation accuracy, while JK-Concat sometimes over-parameterizes the model. JK-LSTM offers a good compromise between expressivity and stability.

Finally, we experiment with using a single readout strategy (mean pooling). While such designs are computationally cheaper, they are less effective. The multi-aggregation strategy, by combining complementary statistical and attention-based summaries, leads to more consistent classification performance.

These architectural decisions reflect a trade-off between model complexity and representational capacity.

### 3.2. Data and Treatments

In order to model AIS reception effectively, we rely on a variety of data sources that together form the integrated AIS dataset. These sources include both raw measurement data and contextual data—each contributing unique and complementary information for the modeling task.

**Figure 3:**
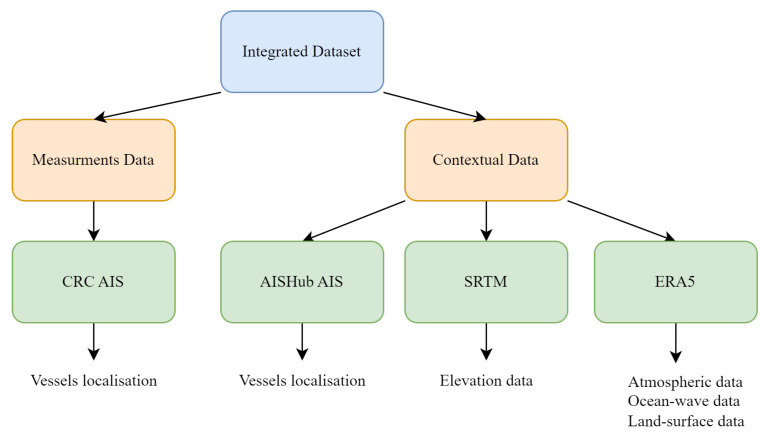
Typology of Data Sources for the integrated AIS reception dataset.

Figure 3 presents an overview of the data used to construct the integrated AIS reception dataset. The figure distinguishes between measurement data—produced by the Centre for research on Risks and Crises (CRC) of Mines Paris–PSL—and contextual data—obtained from external sources—detailing the origins and types of extracted data for each. Measurement data includes AIS messages such as MMSI, position and timestamp. Contextual data includes sources like ERA5, AIShub and SRTM—providing atmospheric, ocean-wave, land-surface and elevation data, which are used to enrich the reception modeling process.

In the following sections, we describe in more detail the different data components used in the modeling workflow. Section 3.2.1 focuses on the measurement data, including AIS message ingestion, processing architecture and storage strategy. Section 3.2.2 introduces the contextual datasets and outlines the preprocessing steps applied to harmonize them with the AIS data. Finally, Section 3.2.3 presents how datasets are combined to build an integrated AIS reception dataset.

#### 3.2.1. Measurement Data

Since August 2018, the CRC laboratory has been collecting NMEA AIS frames transmitted by vessels in the Mediterranean Sea. This dataset includes up to 1 million frames per day. For this study, we retain only AIS Class A data containing georeferenced vessel locations [76].

The acquisition of these NMEA frames is performed by a sensor composed of a WY 155-3N YAGI antenna (manufactured by Sirio Antenne, Volta Mantovana (MN), Italy) installed at an altitude of 188 m above sea level. The antenna has a directional axis of 100 degrees and a beamwidth of 130 degrees. It is connected via an ULTRAFLEX 10 coaxial cable (manufactured by Messi & Paoloni, Ancona (AN), Italy) to a SLR350N AIS receiver (manufactured by Comar Systems, Newport, Isle of Wight, UK). The receiving station is geographically located in Sophia Antipolis, in the southeast of France, near the Mediterranean coast. The specifications of this setup are detailed in Table A1, Table A2, Table A3.

Once the acquisition system is connected to the local network, the AIS frames are timestamped and stored.

The raw AIS data collected by the CRC station is thus composed of millions of NMEA-formatted messages per day. This volume and the need for multiple stages of quality control and transformation necessitate the adoption of a robust and modular data architecture.

To ensure data reliability, traceability and reusability across experiments, we adopt a medallion architecture, a design pattern that is increasingly used in large-scale data processing pipelines [77,78,79]. This architecture organizes data into three layers—Bronze, Silver and Gold—each corresponding to a level of refinement and validation:

- The Bronze layer stores raw, unfiltered AIS frames as collected from the station in log files, preserving original information and timestamping.
- The Silver layer contains parsed and structured data where AIS messages are decoded, subsampled, types are filtered and basic cleaning (e.g., removal of malformed or incomplete frames) is applied. These data are stored in a TimescaleDB database [80] for optimized query.
- The Gold layer holds a curated and enriched dataset in monthly CSV files, such as downsampled positions and data pre-filtered for modeling tasks.

This structured approach enables the reproducibility of data workflows, the isolation of errors and efficient reprocessing, while maintaining flexibility for further enrichment steps [81].

**Figure 4:**
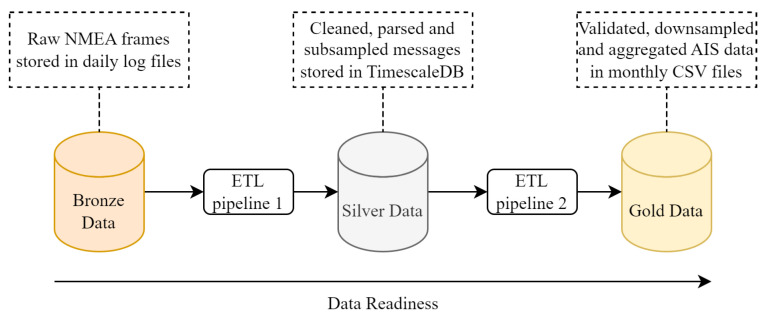
Medallion architecture and ETL pipelines for processing AIS messages. The pipeline processes raw AIS frames through three refinement stages—Bronze (raw), Silver (cleaned, parsed and subsampled) and Gold (validated, downsampled and aggregated).

**Figure 5:**
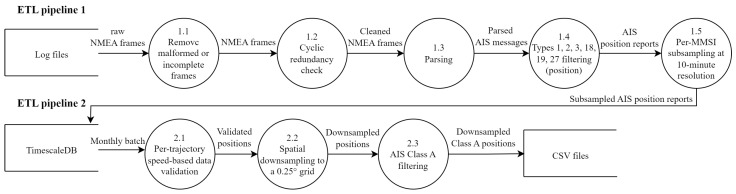
Overview of the ETL pipelines for AIS data processing. The first pipeline (**up**) transforms raw ingested data from the Bronze layer to the Silver layer through cleaning, parsing, filtering and subsampling steps. The second pipeline (**down**) performs advanced transformations from the Silver layer to the Gold layer, including data validation, downsampling and filtering, preparing the dataset for modeling.

To manage the movement of data between layers, we implemented ETL (Extract, Transform, and Load) pipelines as described in Figure 4. These pipelines carry out a series of operations, which are visually represented in the data flow diagrams [82] shown in Figure 5.

#### 3.2.2. Contextual Data

In addition to AIS message data, we integrate several environmental data sources to contextualize vessel movements and support reception modeling. These datasets provide complementary information such as meteorological conditions and topography, which influence maritime navigation behaviors and signal reception conditions.

##### AISHub Data

We supplement our local AIS dataset with messages retrieved from the AISHub platform [83]. These messages follow the same NMEA 4.10 encoding standard and are subjected to equivalent preprocessing steps to those of our in-house data. The ingestion pipeline includes the parsing of raw strings, downsampling and cleaning routines to remove malformed or incomplete records through our ETL pipelines and medallion architecture. Prior to storage, we also performed a filtering step to exclude messages outside our area of interest—the Mediterranean Sea. In terms of volume, the AISHub feed contributes approximately to 40 million messages per day for this area.

##### ERA5 Atmospheric Reanalysis Data

To characterize the environmental context of AIS message propagation, we rely on the ERA5 reanalysis datasets provided by the Copernicus Climate Change Service (C3S) [84]. Two complementary datasets are used:

- ERA5 hourly data on single levels from 1940 to the present [85];
- ERA5 hourly data on pressure levels from 1940 to the present [86].

These datasets offer gridded estimates of meteorological variables with the hourly temporal resolution and spatial resolution of 0.25° × 0.25°, enabling the fine-scale modeling of atmospheric conditions affecting AIS signal propagation.

From the single-level dataset, we extract surface and near-surface variables that are relevant to ducting conditions and maritime atmospheric modeling as well as over-land propagation [87]:

- Wind and temperature: Zonal wind at 10 m, meridional wind at 10 m, air temperature at 2 m, dew point temperature at 2 m.
- Pressure: Surface pressure, mean sea level pressure.
- Refractivity and ducting metrics: Height of the duct base, mean vertical gradient of refractivity inside the trapping layer, minimum vertical gradient of refractivity inside the trapping layer, base height of the trapping layer, top height of the trapping layer.
- Ocean and wave indicators: Mean wave direction, mean wave period, maximum individual wave height, significant height of combined wind waves and swell.
- Precipitation and land surface: Total precipitation, precipitation type, sea surface temperature.
- Vegetation cover and LAI: High vegetation cover, low vegetation cover, leaf area index of high vegetation, leaf area index of low vegetation, type of high vegetation, type of low vegetation.

Sharp vertical gradients in refractivity can lead to atmospheric ducts, which are captured by ERA5 variables such as the trapping layer base and refractivity gradients [17,19]. These variables, together with vertical profiles of temperature and humidity, form a group of parameters related to tropospheric propagation mechanisms, including ducting and scattering due to refractive index variations.

A second group of variables is related to hydrometeor-induced attenuation, including total precipitation, precipitation type and cloud or water vapor content. These parameters are essential to account for signal interference under heavy weather or storm conditions [24,88].

A third category concerns surface interaction effects, including vegetation cover, the leaf area index (LAI) and terrain types. These land surface descriptors help characterize clutter, near-ground diffraction and local signal blocking in coastal or inland areas [89].

Finally, oceanographic variables such as mean wave direction and significant wave height are included to represent sea surface interaction and multipath propagation effects. These can lead to constructive or destructive interference depending on surface state and are especially relevant when modeling AIS signal reflection and scattering over the sea [21,22,90].

The pressure-level dataset is used to reconstruct vertical atmospheric profiles. We extract temperature and relative humidity at the following pressure levels (in hPa):

500,550,600,650,700,750,775,800,825,850,875,900,925,950,975,1000

These variables are critical for evaluating elevated ducting and refractivity profiles of radio refractive index gradiant $\frac{dN}{dz}$ [16,91] which enables the classification of refractive conditions [18,20,24,92].

##### SRTM Elevation Data

Topographical data were extracted from the Shuttle Radar Topography Mission (SRTM) dataset [93], which offers near-global elevation measurements. The native resolution of SRTM is approximately 30 m. To ensure compatibility with ERA5’s resolution, we performed spatial downscaling to a 0.25° × 0.25° grid using resampling. For each grid cell, we computed summary statistics including mean, standard deviation, minimum and maximum elevation values. To handle missing elevation data we used the SRTM Void Filled dataset [94]. These variables serve as static geographical descriptors and can help explain variations in AIS message reception or vessel trajectories near coastlines and mountainous regions.

#### 3.2.3. Integrated AIS Reception Dataset

To enable supervised learning and spatio-temporal modeling, AIS data must be aligned with environmental variables and structured on a common reference grid. We construct an integrated dataset by combining AIS measurements and environmental descriptors on a hourly time step and a spatial grid of resolution 0.25° × 0.25°, consistent with ERA5 reanalysis products. This early node-level fusion allows the direct integration of all descriptors into a unified representation [95]. Several studies demonstrate the benefits of this strategy compared to late fusion, improving both performance and the robustness to noise [96,97,98]. For supervised learning, this choice is decisive: it exposes cross-modal interactions from the earliest layers of the model, providing the classifier with more discriminative information than if each modality were processed independently [96].

Each grid cell at a given time step contains a data vector composed of SRTM-derived, base station and environmental features.

##### Data Vector Structure

**Figure A1:**
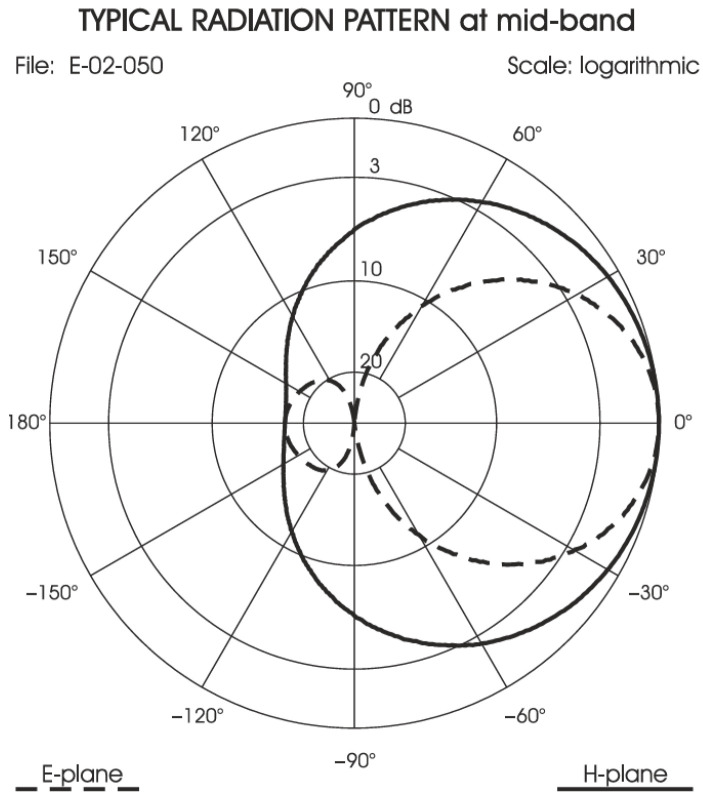
Directivity diagram of the CRC station’s antenna (figure from Sirio Antenne’s documentation—reproduced with permission).

**Figure A2:**
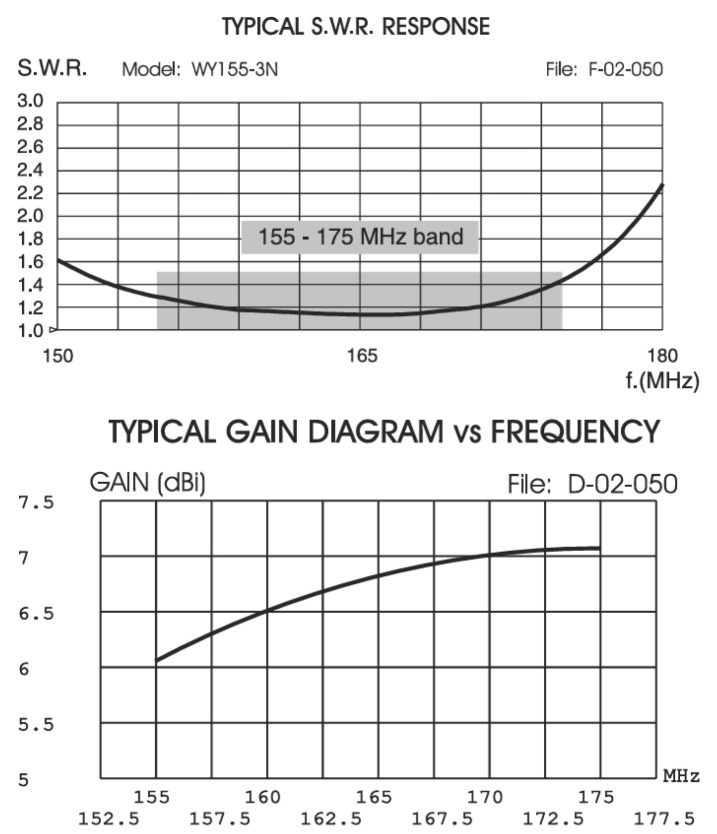
Gain curve of the CRC station’s antenna as a function of frequency (figure from Sirio Antenne’s documentation—reproduced with permission).

For each spatial cell and hourly time slot, we construct a vector with the following features:

- Base station features: Gain associated with the antenna radiation pattern (as derived from Figure A1 and Figure A2) and the distance between the base station and each vessel;
- Atmospheric features: ERA5 Atmospheric data from Section 3.2.2;
- Topographical features: SRTM-derived statistics from Section 3.2.2.

**Figure 6:**
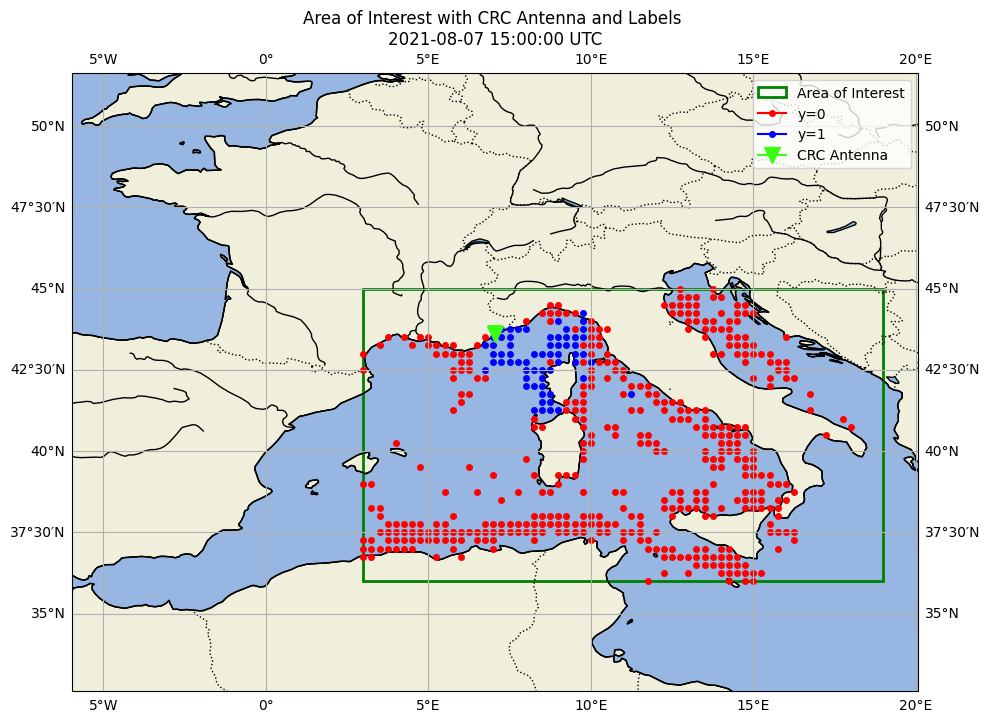
Example of a labeled spatial grid for a single hourly time step. Each cell contains a label: 1 (received locally), 0 (received via AISHub only).

Each data vector is generated for individual grid cells within a bounded spatial domain covering the northwestern Mediterranean region as shown in Figure 6. It is defined over the following latitude and longitude ranges (see Equation (4)):

(4) $$\begin{array}{cccc} {\mathrm{LAT}}_{\min} & =36^{\circ }, & \;{\mathrm{LAT}}_{\max} & =45^{\circ }\\ {\mathrm{LON}}_{\min} & =3^{\circ }, & \;{\mathrm{LON}}_{\max} & =19^{\circ } \end{array}$$

##### Supervised Labeling

For each time step of one hour, a full grid was generated over the study area, and we assigned to each cell across the entire grid a label, based on vessel presence, with the following values:

- A value of 1 indicates that at least one vessel was detected within the cell and successfully received by our local AIS station.
- A value of 0 indicates that a vessel was present in the cell according to the AISHub dataset, but it was not received by our local station.
- A value of −1 denotes that no vessel has been detected in the cell by either our local antenna or any AISHub antenna during that time step.

This labeling strategy supports supervised learning tasks by providing a binary classification target (received vs. not received) with masked cells (−1) excluded from the training set. It allows models to learn reception conditions based on spatial, temporal and environmental features associated with each labeled cell. Figure 6 illustrates an example of the labeled spatial grid at a given hourly time step. Each grid cell is represented by a central point, color-coded according to its AIS reception label, green for positive reception (label 1) and red for no reception (label 0). Cells with label −1 are not shown.

### 3.3. Graph Construction and Feature Engineering

Each node in the graph corresponds to a cell in the hourly spatial grid introduced in Section 3.2.3. These grid cells are defined at a fixed spatial resolution (0.25° × 0.25°) and repeated for every hourly time slot. The feature vector associated with each node includes all relevant environmental descriptors extracted from the data vector described previously. These features are computed per cell and per time slot and are assumed to be aligned and synchronized with the AIS reception data [99].

Graphs are generated separately for each hourly time slot. For each such grid, we process the associated node set to build multiple directed graphs. Specifically, we generate one graph per node labeled with a class value different than −1. Each labeled node (with label 0 or 1) is considered a transmitter and a graph is constructed to model signal propagation from that Tx node toward a fixed Rx node representing the AIS base station [56].

To construct these graphs, we define a message-passing structure based on the local grid topology. Starting from the Tx node, we iteratively connect it to its Moore neighborhood (those within a Chebyshev distance ${∥\;\cdot \;∥}_{\infty }=1$). This process continues recursively, connecting neighbors to their neighbors, until the Rx node is reached. The resulting graph is directed, with edges following the propagation direction from Tx to Rx. To restrict the spatial extent of propagation we use a cone-based filtering approach.

We also define the directional cone as the geometric region oriented along the Tx–Rx axis within which propagation paths are considered, and we define the cone aperture α (a model hyperparameter) as the full angle of the directional cone. Only nodes falling within this cone are retained in the final graph.

The complete procedure for graph generation is summarized in Algorithm 1.
**Algorithm 1** Graph construction from labeled grid cells**Require:  **Grid $G_{t}$ for time slot *t*, labels *L*, receiver location Rx, cone aperture α  1:**for all** cell $v_{i}\in G_{t}$ **do**  2:    **if** $L[v_{i}]\in 0,1$ **then**  3:         Set $v_{i}$ as transmitter (Tx)  4:         Initialize graph $G_{i}$ with $v_{i}$ as source node  5:         Initialize queue $Q\leftarrow v_{i}$  6:         **while** *Q* not empty and Rx not reached **do**  7:             Pop $v_{j}$ from *Q*  8:             **for all** neighbor $v_{k}$ of $v_{j}$ in Moore neighborhood **do**  9:                 **if** $v_{k}$ lies within cone of aperture α centered at Tx **then**10:                     Add edge $v_{j}\to v_{k}$ to $G_{i}$11:                     Add $v_{k}$ to *Q* if not already visited12:                 **end if**13:             **end for**14:         **end while**15:         Store $G_{i}$ with label $L[v_{i}]$16:    **end if**17:**end for**

**Figure 7:**
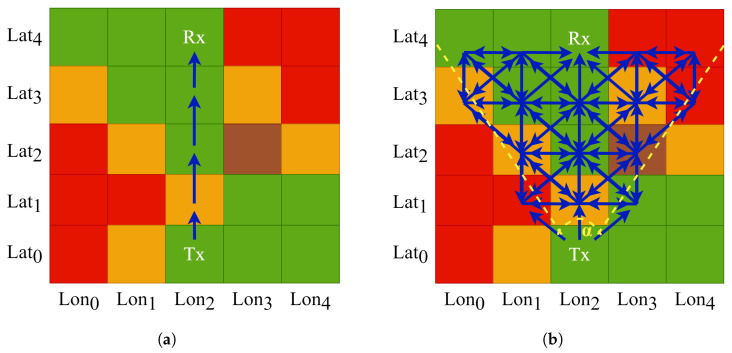
Comparison between propagation modeling strategies from a transmitter (Tx) to a fixed receiver (Rx) based on propagation conditions (green: good; orange: medium; red: bad; brown: terrain effects). (**a**) Naïve direct propagation. (**b**) Complex propagation constrained by a directional cone.

This procedure ensures that each graph corresponds to a plausible propagation scenario associated with a labeled grid cell. The use of a directed structure encodes the directional nature of radio wave propagation; however, the graph is not necessarily acyclic, as bidirectional edges may exist depending on the spatial configuration. The angular filtering controls graph sparsity and orientation [100]. Figure 7 illustrates the evolution from a naive grid-based approach to the structured graph modeling strategy adopted in this work.

Figure 7a shows the LoS propagation model explored in our previous study [52] and test cases, where the path from the transmitter (Tx) to the receiver (Rx) is defined as a straight vector traversing all intersected grid cells. While simple to implement, this approach disregards local spatial interactions and environmental variability surrounding the main axis of propagation.

The Figure 7b presents our graph-based propagation model, where each node is initially connected to its eight neighbors to enable recursive expansion from Tx to Rx. In the proposed approach, we introduce a directional constraint in the form of a cone with aperture α centered on the Tx–Rx axis. This geometric filter focuses the graph structure on regions that are more likely to contribute to signal propagation, effectively reducing the influence of marginally relevant areas. The aperture α serves as a tunable hyperparameter to balance expressiveness and selectivity.

To reduce heterogeneity in feature magnitudes and facilitate convergence during training, all node features are standardized. We apply min–max normalization, which rescales each feature to the [0,1] interval using the minimum and maximum values observed across the dataset. This approach is particularly suitable for physical variables, which are naturally bounded [101].

## 4. Results

This section presents the experimental evaluation of our proposed model for AIS reception area prediction. We begin by describing and justifying the selection of baseline models used for comparison, ranging from traditional physics-based approaches to machine learning and graph-based methods. We then detail the training setup, including the model hyperparameter configuration. Quantitative results are reported using a variety of performance metrics to assess both predictive accuracy and computational efficiency. These comparisons aim to highlight the benefits and limitations of each modeling approach. Finally, we highlight the limits of our model and provide a discussion of the observed results.

**Figure 8:**
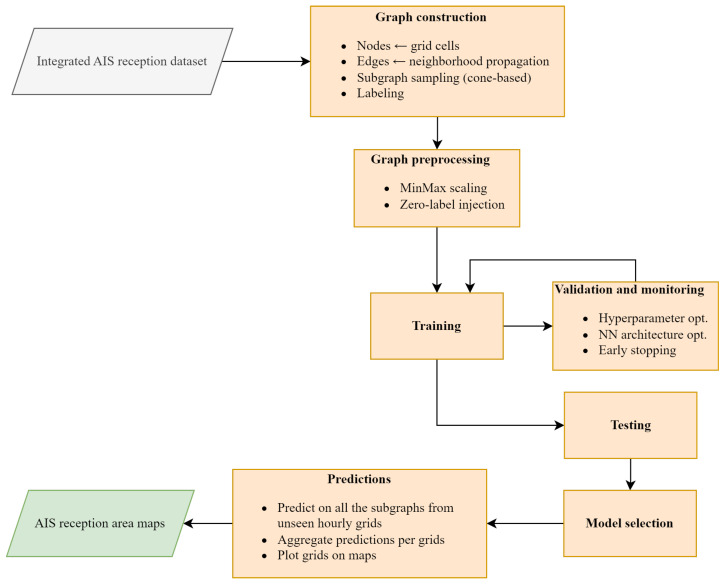
Overview of the training and prediction pipeline.

The full model pipeline is illustrated in Figure 8. The system proceeds from graph construction and feature normalization to model training, validation, evaluation and predictions. Each graph is built around a labeled transmitter node, with edges constrained by a directional cone as described in Section 3.2. Training is performed using binary classification at the graph level, with evaluation metrics computed on held-out test graphs.

### 4.1. AIS Propagation Models

To evaluate the performance of our proposed graph-based model, we compare it against a set of baselines drawn from three categories: traditional physics-based models, classical machine learning algorithms and alternative graph neural network architectures. Each model operates on a different representation of the input data, with distinct biases and limitations. Table 1 summarizes their key characteristics.

#### 4.1.1. ITU-R P.2001: Physics-Based Baseline

The ITU-R P.2001 model [38] serves as a deterministic reference based on physical principles of radiowave propagation. It computes the basic transmission loss between transmitter and receiver using detailed environmental inputs, including terrain profiles, atmospheric refractivity, surface types and antenna characteristics. The method is designed for point-to-point long-range communication and is widely used in engineering contexts. A message is considered received if the predicted power at the receiver exceeds our AIS receiver sensitivity (−112dBm). While grounded in electromagnetic theory, this model is sensitive to noise in input variables and is computationally expensive due to the required digital terrain and climate maps (see Table A4).

#### 4.1.2. XGBoost: Classical Machine Learning Baseline

XGBoost [102] is a tree-based ensemble method that has demonstrated strong performance in various tabular learning tasks. In our case, the model takes as input a set of environmental and geographic features extracted for each Tx–Rx pair. These features are aggregated into a vector on the straight line propagation path. While XGBoost is fast and interpretable, it lacks the ability to model graph structures.

#### 4.1.3. Graph Convolutional Network (GCN)

The GCN [103] is a foundational architecture in graph learning, where node features are aggregated from local neighborhoods using fixed-weight averaging. While they are effective for shallow architectures, GCNs are known to suffer from over-smoothing when depth increases [104]. In our implementation, the GCN is configured with five layers and 128 neurons, using ReLU activations. This architecture serves as a baseline to compare graph models.

#### 4.1.4. Graph Attention Network (GAT)

The GAT [64] extends the GCN by learning attention weights over neighboring nodes during aggregation. This enables the model to focus on more informative parts of the graph. For AIS reception, this mechanism can help highlight influential regions along the propagation path. However, GAT introduces heavy computational costs and its performance can degrade in noisy graphs [64,75]. Our implementation uses five layers, 128 neurons and multi-head attention with one head per layer.

### 4.2. Implementation, Training and Validation

This section details the experimental setup used to train and evaluate the proposed GNN-based model for AIS reception prediction. We describe the hardware configuration, software stack, dataset splitting strategy, training protocol and hyperparameter tuning process.

#### 4.2.1. Hardware and Execution Environment

All training and evaluation procedures were conducted on a high-performance computing server equipped with two NVIDIA Quadro RTX 8000 GPUs (2 × 46 GB VRAM), an Intel Xeon Silver 4210R CPU (40 cores, 2.40 GHz base frequency), 376 GB of DDR4 RAM and 11 TB of SSD storage with a read speed of approximately 7212 MB/s. Each complete training cycle for the proposed GNN model took approximately 100 h, including graph generation, feature standardization and training with early stopping.

Inference experiments were conducted on a consumer-grade machine featuring an NVIDIA RTX 3070 GPU (8 GB), an Intel Core i7-10700K CPU (8 cores/16 threads, 3.80 GHz) and 32 GB of RAM. This setup was used to benchmark prediction latency and test performance in conditions that are more representative of common deployment environments.

#### 4.2.2. Software Stack

The model was implemented in Python using PyTorch Geometric version 2.5.3 [105] as the core framework for graph neural network layers and message passing operations. PyTorch version 2.3.1 [106] was used as the tensor computation backend and training workflows were managed using PyTorch Lightning version 2.3.3 [107]. XGBoost baseline was implemented with xgboost Python library version 3.0.3 and Scikit-learn version 1.5.1 [108] used primarily for training and evaluation.

The deterministic physics-based model ITU-R P.2001 was implemented using the Py2001 package version 4.0 [109]. This implementation is a Python translation of the official MATLAB/Octave reference version of the Recommendation [38], as approved by ITU-R Working Party 3M and published by Study Group 3 on the ITU-R SG 3 Software, Data and Validation portal [110].

All experiments were orchestrated through a reproducible training pipeline leveraging PyTorch Lightning’s logging and checkpointing utilities, with deterministic seed initialization to ensure consistency across runs.

#### 4.2.3. Data Partitioning

The dataset covers a four-year period spanning from 2020 to 2023. The study area is geographically bounded by the latitude and longitude limits defined in Equation (4).

For evaluation, the dataset was partitioned into three disjoint subsets: training (95%), validation (5%) and a separate test set. The test set comprises 2602 data points, each extracted from distinct hourly grid instances evenly distributed across the full dataset. This corresponds to 10% of the data prior to the training/validation split. The spatial distribution of the test set was not artificially homogenized; instead, it directly reflects the natural data distribution observed in the study area. This ensures that the evaluation results are representative of real operational conditions, rather than being biased by an enforced uniform sampling.

#### 4.2.4. Training Protocol

Model optimization was performed using the Adam optimizer [111], with a batch size of 32 chosen to balance training stability and computational efficiency given our available computational resources, and early stopping based on the validation metrics and weight checkpoints saved for the top three lowest validation losses. The learning rate was set to $1\times 10^{-3}$. To mitigate intra-grid class imbalance, each data point within a given hourly grid was sampled with equal probability across classes. The full training configuration is summarized in Table 2.

**Figure 9:**
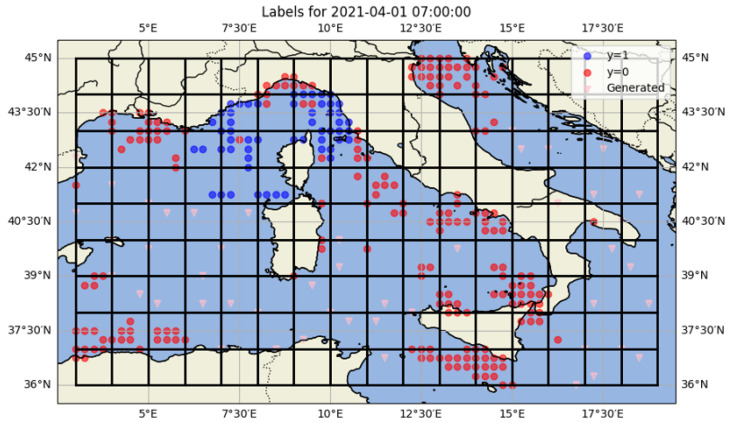
Labeled spatial supplemented with generated 0 labels as described in Algorithm 2. A grid of cells with a size of 0.25° × 4 (data generation block size) is overlaid, ensuring that each cell contains at least one datapoint.

To address the issue of regional imbalance in AIS coverage—particularly in zones with a low density of reception reports due to the lack of AISHub receivers—we implemented a data generation strategy to ensure more uniform spatial coverage. Specifically, we synthetically assigned the label 0 (non-reception) to a small number of randomly chosen grid cells in underrepresented areas (Figure 9). This strategy is designed as a general framework that systematically scans the spatial domain in fixed-size blocks (0.25° × 4). Within each block, if reception is already represented (label 1) or non-reception is already represented (label 0), the block is left untouched. Only in the case where all cells are unlabeled (label −1) does the algorithm randomly assign a single cell as non-reception (0). In this way, the method ensures at least one datapoint per block without artificially inflating dense regions, thereby mitigating the risk of overfitting to heavily monitored zones and improving generalization to sparsely covered regions.

The data generation procedure operates by scanning the entire spatial domain using fixed-size blocks and checking the label distribution within each block. If a block contains only unlabeled cells (label −1), a single cell is randomly selected and labeled as 0. The process is summarized in Algorithm 2.
**Algorithm 2** Spatial zero-label injection for coverage balancing**Require:** Dataset *D* with coordinates and labels *y*, spatial bounds $\left({\mathrm{LAT}}_{\min},{\mathrm{LAT}}_{\max}\right)$, 
       $\left({\mathrm{LON}}_{\min},{\mathrm{LON}}_{\max}\right)$, resolution *r*, block size *N*
  1:**for all** block *B* in *D* with size N×N **do**  2:    Extract labels $\ell_{B}$ of all cells in *B*  3:    **if** 1 $\in \ell_{B}$ and (0 $\in \ell_{B}$ or $-1\in \ell_{B}$) **then**  4:        **continue** {Reception already modeled}  5:    **else if** 0 $\in \ell_{B}$ **then**  6:        **continue** {Non-reception already modeled}  7:    **else if** All $\ell_{B}=-1$ **then**  8:        Randomly select one index i∈B and set $y_{i}\leftarrow 0$  9:    **end if**10:**end for**11:**return** Modified dataset *D*

#### 4.2.5. Hyperparameter Tuning

Hyperparameter selection was carried out through a grid search strategy on the validation set. The parameters explored included the number of hidden dimensions, the number of GNN layers and the dropout rate. In practice, the choice of the final hyperparameters was supported by short simulation runs over a few epochs, where convergence behavior, convergence speed and validation accuracy were systematically monitored. This procedure allowed us to identify the configuration that provided the best balance between stability and predictive performance. Table 3 summarizes the hyperparameter search space and the selected final values for the model.

### 4.3. Performance Comparison and Evaluation

This section presents a comparative analysis based on multiple performance metrics. We also include visualizations to support the interpretation of the results and highlight specific areas.

#### 4.3.1. Evaluation Metrics

We report the following standard classification metrics: accuracy, recall, precision, F1-score. In addition, we include inference time on the test set as an indicator of computational efficiency. These metrics offer complementary perspectives: accuracy captures overall correctness, recall emphasizes the ability of the model to detect true receptions and precision measures false alarm tolerance. These metrics are weighted averages.

#### 4.3.2. Proposed GNN Predictions

To examine the behavior of the proposed GNN model, we present the confusion matrix in Table 4. Out of 2602 test samples, the model correctly identifies 1484 true reception cases and 1006 true non-reception cases. Only 25 reception cases are missed (false negatives) and 87 cases are incorrectly predicted as reception (false positives). This indicates a high true-positive rate and a low false-positive rate, consistent with the strong F1-score.

**Figure 10:**
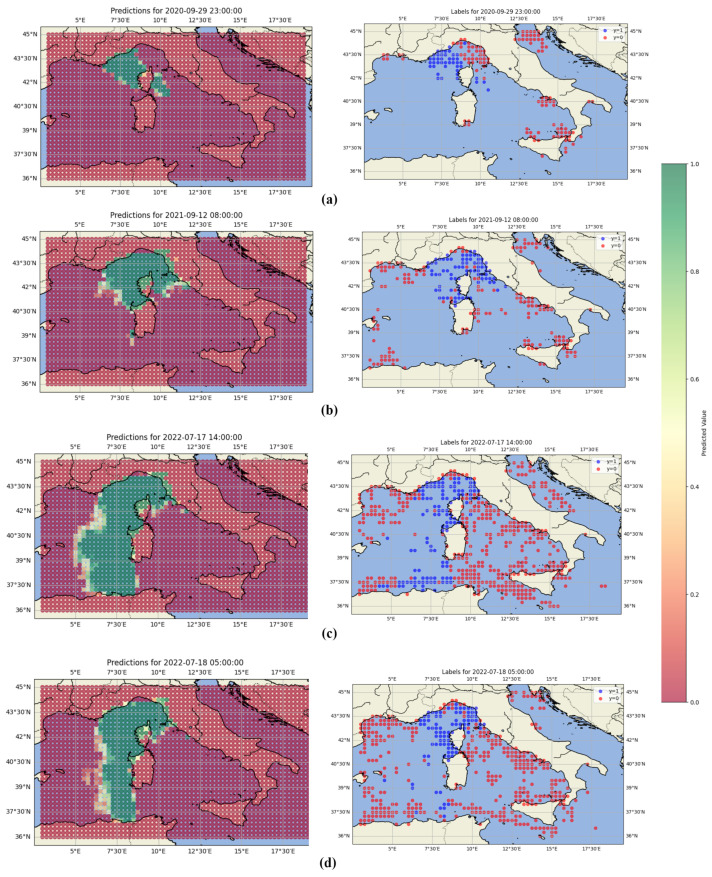
Visual validation and comparison of model predictions for different scenarios. (**a**) corresponds to a short-range reception scenario, with some receptions observed behind Corsica. (**b**) represents cases with receptions located further east, near the Italian coast. (**c**,**d**) correspond to long-range reception scenarios, where the signals originate near the African coasts, with (**d**) being narrower.

Figure 10 illustrates typical predictions made by the proposed model for four contrasting reception scenarios. Right maps represent the ground truth labels, while left maps show the model’s predictions. The two top pairs ((a) and (b)) correspond to short-range reception cases where the predicted signal footprint is compact and close to the base station. In contrast, the bottom ((c) and (d)) pairs depict long-range scenarios in which the model identifies an extended reception area, consistent with potential atmospheric effects such as ducting.

To generate such prediction maps, the model is applied to all graphs constructed on the underlying spatial grid. Each graph corresponds to a candidate transmission cell and the predicted label is used to reconstruct the spatial reception area by aggregating the outputs across the grid.

#### 4.3.3. Performances

Table 5 summarizes the performance of all models on the test set. The proposed GNN model achieves the highest scores across all evaluation metrics: accuracy, recall, precision and F1-score. With an F1-score of 0.957, it demonstrates a strong ability to correctly classify both reception and non-reception zones, outperforming the second-best model (GAT) by more than 2.5 percentage points.

In terms of test time, the GCN model is marginally the fastest (54.73 s), followed closely by GAT and XGBoost. The proposed model completes inference over the 2602 test samples in 57.73 s, which remains competitive and well-suited for operational use.

These results confirm that our graph-based approach offers a favorable trade-off between predictive performance and computational efficiency, making it suitable for real-time maritime situational awareness applications. In practice, using the consumer-grade machine, the model is able to predict an entire AIS reception area—composed of 2405 individual graph classifications—under a given environmental condition in less than 10 s.

**Figure 11:**
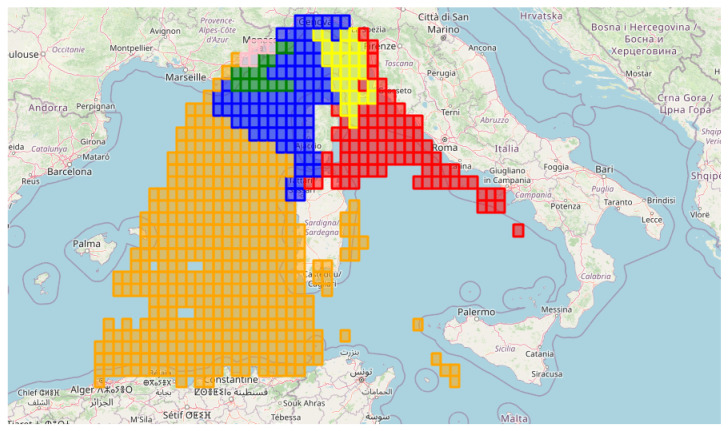
Spatially coherent maritime sub-regions obtained through spatio-temporal hierarchical clustering of grid cells based on reception time series and spatial adjacency.

To address the potential concern of spatial leakage due to the temporal train/test split, we further refine the performance analysis through a spatio-temporal clustering procedure. Each grid cell is encoded as a temporal binary sequence of reception/non-reception over the three-year period, and a connectivity matrix is built considering the spatial neighborhood (eight directions). Hierarchical clustering with Ward’s linkage [112] was then applied under this spatial constraint, yielding geographically coherent clusters. These correspond to identifiable maritime sub-regions (Figure 11).

The clusters are of heterogeneous size: Cluster 0 (Blue, 606 samples), Cluster 1 (Green, 385 samples), Cluster 2 (Yellow, 332 samples), Cluster 3 (Red, 542 samples), Cluster 4 (Pink, 298 samples), and Cluster 5 (Orange, 439 samples). This distribution highlights both densely and sparsely represented maritime sub-regions, which is important to interpret the performance metrics reported below.

The results, compared between XGBoost and the proposed model, in order to support a graph-based approach rather than a vector-based one, are summarized in Table 6, but the most contrasted clusters can be highlighted as follows:

In the Gulf of Genoa (Cluster 2—Yellow), the proposed GNN maintained balanced performance, with a precision and recall of 0.904 for the reception class (1). By contrast, XGBoost showed an imbalance: although its recall for class 1 was slightly higher (0.923), it suffered from a poor detection of class 0 (recall =0.702), resulting in weaker F1-scores overall. This indicates that the proposed model provides more stable predictions across both classes in this region.

In the Tyrrhenian Basin (Cluster 3—Red), both models reached high performance, but the proposed GNN stood out on class 1 with a recall of 0.942 and an F1-score of 0.905, compared to 0.884 and 0.835 for XGBoost. This confirms its stronger detection capability for the reception class in this dense observation area.

In all other regions, both models performed evenly, with perfect scores in Clusters 1 and 4, and only marginal differences elsewhere.

These results show that the proposed model consistently enhances the detection of the reception class while preserving balanced performance across classes and maritime sub-regions, thereby mitigating the risk that global scores are artificially inflated by spatially adjacent cells appearing in both training and test sets.

## 5. Discussion

While the proposed GNN-based model demonstrates strong performance for AIS reception prediction, several limitations remain, both in the design of the methodology and in the scope of the evaluation.

### 5.1. Methodological Limitations

One limitation stems from assumptions made during data preprocessing and graph construction. The model relies on a fixed spatial grid of 0.25° × 0.25° resolution, which may be too coarse to capture small-scale variations in terrain or atmospheric properties that affect signal propagation. Furthermore, the directional cone used to limit the graph neighborhood is a manually selected hyperparameter, which may not fully reflect the complexity of real-world propagation dynamics. Although this filtering improves computational cost and aligns with physical heuristics, it introduces an artificial boundary that may exclude relevant spatial interactions.

### 5.2. Model Limitations

From a modeling perspective, the current architecture captures spatial structure but does not explicitly model temporal dynamics. All graphs are constructed independently for each hourly time slot, without considering the continuity or evolution of environmental conditions. This could limit the model’s ability to capture persistent propagation patterns. Additionally, while the multi-aggregation readout improves the expressive power of the model, it increases the number of parameters.

### 5.3. Evaluation Limitations

Our evaluation is performed on a geographically bounded dataset. Although we applied inductive graph representation learning, the generalization capability of the model to entirely unseen maritime regions remains to be validated. Some baselines, such as ITU-R P.2001, are also highly dependent on the quality of auxiliary data, which may not be uniformly available or accurate across all regions. In addition, the ground truth labels used for training and evaluation are derived from AIS reception logs, which are themselves subject to errors. In regions with sparse vessel traffic or poor AISHub coverage, non-reception may reflect a lack of emitters rather than true signal attenuation, potentially introducing ambiguity in the classification task.

### 5.4. Resource and Practical Constraints

Several practical constraints limited the scope of this study. First, the collection and alignment of environmental data, particularly from meteorological reanalyses, require significant storage and preprocessing time. Second, high-resolution graph construction and GNN training are computationally intensive, constraining the number of model variants and repetitions explored. Finally, AIS reception data is inherently noisy. Variability in vessel transmission behavior, hardware inconsistencies, and antenna misalignment can all contribute to signal dropouts that are unrelated to propagation conditions.

## 6. Conclusions

In this work, we proposed a graph-based modeling approach for predicting AIS reception areas from AIS and environmental data. Leveraging the expressive power of GNNs, our method constructs spatially grounded graphs from gridded hourly observations and classifies each subgraph using a deep architecture combining GraphSAGE convolution, LSTM-based JK and a multi-aggregation readout module. This design enables the model to learn both local and global reception patterns, while doing inductive representation learning.

We benchmark our approach against several baselines, including the deterministic ITU-R P.2001 model, a machine learning method (XGBoost) and alternative GNN architectures (GCN, GAT). The proposed model consistently achieved the best F1-score, demonstrating a strong balance between recall and precision. Its superiority was especially notable in non-LoS regions, highlighting its capacity to detect long-range reception events that are potentially linked to ducting or anomalous propagation.

Beyond raw performance, this study also introduces a methodology for environmental graph construction and evaluation. It also accounts for physical constraints through directional filtering. The experimental pipeline is designed to reflect realistic maritime monitoring scenarios, using real AIS data across four years and leveraging high-resolution terrain and reanalysis products. To support this pipeline, we adopt a medallion architecture—organizing data into raw, cleaned and ML-ready stages—which enabled scalable ETL processes, reproducibility and the integration of heterogeneous inputs.

Yet, several limitations remain. The ground truth labels are inferred from AIS reception data, which may include noise and coverage bias due to variable transmission activity. The current model is spatially structured but temporally agnostic and relies on static cone-based neighborhood definitions. Evaluation was conducted in a geographically bounded setting, constrained by the coverage area of our self-owned and self-operated AIS receiver antenna. As a result, generalization to open-ocean or low-traffic regions remains to be validated, particularly in areas not represented in the training dataset.

Future work could explore the integration of temporal information through recurrent or temporal GNN architectures [66,113,114], allowing the model to capture evolving atmospheric conditions. Data augmentation strategies, such as synthetic trajectory generation [115], may improve robustness. Transfer learning or domain adaptation approaches could also be beneficial for extending the model to new maritime regions without retraining from scratch. To overcome limitations in coastal receiver coverage, integrating Satellite AIS (S-AIS) [1,116] data could provide a more comprehensive view of AIS reception and enable training on sparsely monitored areas.

Overall, this work highlights the effectiveness of a data-driven, graph-based methodology for modeling radio wave reception in maritime environments. By leveraging graph construction, environmental feature integration and deep learning, our approach provides a flexible and scalable framework for AIS coverage prediction. It demonstrates how graph neural networks can capture real-world reception behavior directly from observational data, offering new perspectives for ship tracking.

## Figures and Tables

**Table 1:** Summary of baseline and proposed models for AIS reception prediction.

Model	Input Structure	Spatial Modeling	Type
ITU-R P.2001	See Table A4 + charts [38]	Analytical propagation path	Physics-based
XGBoost	Tabular features	Straight line vector	ML
GCN	Graph (nodes + edges)	Fixed neighborhood aggr.	GNN
GAT	Graph (nodes + edges)	Attention-based aggr.	GNN
Proposed GNN	Graph (nodes + edges)	Hierarchical aggr.	GNN

**Table 2:** Summary of training configuration and hyperparameters used for model optimization.

Component	Setting
Optimizer	Adam
Learning rate	$1\times 10^{-3}$
Batch size	32
GNN layers	5
MLP layers	2
Hidden dimension	128
Activation function	ReLU
Last activation function	Sigmoid
Loss function	Binary Cross-Entropy
Directional cone aperture	30
Data generation block size	4

**Table 3:** Hyperparameter search space used during model tuning.

Hyperparameter	Range Tested	Final Value
Learning rate	$\left\{1\times 10^{-2},1\times 10^{-3},1\times 10^{-4},1\times 10^{-5}\right\}$	$1\times 10^{-3}$
Hidden dimension	{64, 128, 256, 512}	128
Number of GNN layers	{3, 5, 7}	5
Number of MLP layers	{2, 4}	2
Directional cone aperture	{15, 30, 60, 90, 120}	30
Data generation block size	{None, 3, 4}	4

**Table 4:** Confusion matrix for the proposed GNN model on the test set. TN: True Negative; FP: False Positive; FN: False Negative; TP: True Positive. 0: No Reception, 1: Reception.

	0 (Predicted)	1 (Predicted)
**0 (Observed)**	1006 [TN]	87 [FP]
**1 (Observed)**	25 [FN]	1484 [TP]

**Table 5:** Overall performance metrics for all compared models on the test set with 2602 samples (best values in bold).

Model	Accuracy	Recall	Precision	F1-Score	Test Time (s)
ITU-R P.2001	0.826	0.826	0.839	0.827	60.45
XGBoost	0.916	0.916	0.916	0.916	56.93
GCN	0.918	0.918	0.920	0.918	**54.73**
GAT	0.932	0.932	0.932	0.932	55.71
Proposed GNN	**0.957**	**0.957**	**0.958**	**0.957**	57.73

**Table 6:** Detailed performance metrics per class (0 and 1) for each cluster and model on the test set (best values in bold).

Cluster	Model	Precision		Recall		F1-Score	
		0	1	0	1	0	1
Cluster 0 (Blue)	Proposed GNN	0.853	**0.950**	**0.777**	0.970	**0.813**	**0.960**
	XGBoost	**0.866**	0.922	0.634	**0.978**	0.732	0.949
Cluster 1 (Green)	Proposed GNN	1.000	1.000	1.000	1.000	1.000	1.000
	XGBoost	1.000	1.000	1.000	1.000	1.000	1.000
Cluster 2 (Yellow)	Proposed GNN	0.839	**0.904**	**0.839**	0.904	**0.839**	**0.904**
	XGBoost	**0.845**	0.838	0.702	**0.923**	0.767	0.879
Cluster 3 (Red)	Proposed GNN	**0.989**	**0.871**	**0.974**	**0.942**	**0.981**	**0.905**
	XGBoost	0.978	0.792	0.956	0.884	0.967	0.835
Cluster 4 (Pink)	Proposed GNN	1.000	1.000	1.000	1.000	1.000	1.000
	XGBoost	1.000	1.000	1.000	1.000	1.000	1.000
Cluster 5 (Orange)	Proposed GNN	**0.968**	**0.879**	**0.990**	0.691	**0.979**	**0.773**
	XGBoost	0.968	0.829	0.985	0.691	0.976	0.753

## Data Availability

Processed AIS data used for modeling will be made available by the authors on request.

## Appendix A

### Appendix A.1. Acquisition Setup

**Table A1:** Specifications of the CRC station’s antenna (from Sirio Antenne’s documentation).

Parameter	Value
Type	3-element Yagi
Frequency range at VSWR ≤1.5	155–175 MHz
Horizontal beamwidth at −3 dB	130°
Vertical beamwidth at −3 dB	70°
Gain	see Figure A1 and Figure A2

**Table A2:** Signal loss for the CRC station’s cable (from Messi & Paoloni’s documentation).

Frequency (MHz)	Attenuation (dB/100 m)
144	7.40
200	8.90

**Table A3:** Specifications of the AIS receiver at the CRC station (from Comar Systems’ documentation).

Parameter	Value
Frequencies	161.975 MHz and 162.025 MHz
Sensitivity	−112dBm

### Appendix A.2. Physics-Based Model Configuration

**Table A4:** Input parameters required by the ITU-R P.2001 propagation model, with values used in our implementation.

Variable	Type	Unit	Description	Value
d	array double	km	Terrain profile distances	SRTM
h	array double	m (asl)	Terrain profile heights	SRTM
z	array int	–	Zone code (1: Sea, 3: Coastal, 4: Inland)	SRTM
GHz	scalar double	GHz	Frequency (0.3–50 GHz)	0.165
Tpc	scalar double	%	Time percentage for loss not exceeded	–
Phire	scalar double	deg	Rx longitude (east positive)	7.0
Phirn	scalar double	deg	Rx latitude (north positive)	43.5
Phite	scalar double	deg	Tx longitude (east positive)	–
Phitn	scalar double	deg	Tx latitude (north positive)	–
Hrg	scalar double	m	Rx antenna height above ground	188
Htg	scalar double	m	Tx antenna height above ground	15
Grg	scalar double	dBi	Rx antenna gain (toward Tx)	Figure A1
Gtg	scalar double	dBi	Tx antenna gain (toward Rx)	5
FlagVP	scalar int	–	Polarization (1: vertical, 0: horizontal)	0

For Tpc, we derive ducting probability from ERA5 atmospheric reanalysis data on single levels using the duct base height variable. Specifically, we compute the proportion of time within a given hour where the duct base height is below a threshold altitude. This value is then used as a proxy for the percentage of time during which anomalous propagation conditions are likely to occur, thereby serving as a physically grounded estimate of Tpc for use in the ITU-R P.2001 model.
